# A recurrent missense mutation in *GJA3* associated with autosomal dominant cataract linked to chromosome 13q

**Published:** 2011-08-20

**Authors:** Thomas M. Bennett, Alan Shiels

**Affiliations:** 1 Department of Ophthalmology and Visual Sciences, Washington University School of Medicine, St Louis, MO; 2 Department of Genetics, Washington University School of Medicine, St Louis, MO

## Abstract

**Purpose:**

To map and identify the genetic defect underlying autosomal dominant cataract segregating in a 5-generation Caucasian American family.

**Methods:**

Genomic DNA was prepared from blood leukocytes, genotyping was performed using microsatellite markers, and logarithm of the odds (LOD) scores were calculated using the LINKAGE programs. Mutation profiling was performed using direct exon cycle-sequencing and restriction fragment analysis. Protein function effects were evaluated using in silico prediction algorithms.

**Results:**

Significant evidence of linkage was obtained at marker D13S175 (maximum LOD score [Z_max_]=3.67; maximum recombination fraction [θ_max_]=0.04) and D13S1316 (Z_max_=2.80, θ_max_=0.0). Haplotyping indicated that the disease lay in the ~170 Kb physical interval between D13S1316 and D13S175, which contained the gene for gap-junction protein alpha-3 (*GJA3*) or connexin-46. Sequencing of *GJA3* detected a heterozygous transition (c.130G>A) in exon-2 that resulted in gain of an Hsp92 II restriction site. Allele-specific PCR amplification and restriction analysis confirmed that the novel Hsp92 II site co-segregated with cataract in the family but was not detected in 192 normal unrelated individuals. The c.130G>A transition was predicted to result in a non-conservative substitution of valine-to-methionine at codon 44 (p.V44M) with damaging effects on protein function.

**Conclusions:**

These data confirm *GJA3* as one of the most frequently mutated genes that underlie autosomal dominant cataract in humans, and further emphasize the importance of connexin function in maintaining lens transparency.

## Introduction

Inherited forms of cataract(s) constitute a clinically heterogeneous disorder of the ocular lens that usually present with an early-onset ranging from birth (congenital) through infancy into the fourth decade (Online Mendelian Inheritance in Man; OMIM). Congenital and infantile forms of cataract that cause blurring of images on the immature retina are a clinically important cause of impaired form vision development (deprivation amblyopia), and pediatric cataract surgery is associated with increased risk of aphakic glaucoma and lifelong visual impairment [1-3].

In addition to being found as a secondary feature of many genetic syndromes and metabolic disorders involving other ocular and/or systemic abnormalities (OMIM), cataract may be inherited as a primary or isolated lens phenotype [4,5]. All three classical forms of Mendelian inheritance have been described. However, most families reported exhibit autosomal dominant transmission with high penetrance. So far genetic linkage studies of around 180 families worldwide have mapped at least 35 independent loci and identified mutations in over 20 genes for phenotypically diverse forms of primary cataract involving total, nuclear, lamellar/zonular, sutural, and polar/sub-capsular lens opacities [6].

Approximately 55% of the known mutations underlying inherited forms of primary cataract have been detected in ten crystallin genes; alphaA-crystallin (*CRYAA*), alphaB-crystallin (*CRYAB*), betaB1-crystallin (*CRYBB1*), betaB2-crystallin (*CRYBB2*), betaB3-crystallin (*CRYBB3*), betaA1-crystallin (*CRYBA1*), bataA4-crystallin (*CRYBA4*), gammaC-crystallin (*CRYGC*), gammaD-crystallin (*CRYGD*), and gammaS-crystallin (*CRYGS*) that encode the major “refractive” proteins of the lens [7-15]. A further 20–25**%** of known mutations have been detected in two genes encoding gap-junction protein alpha 3 and alpha 8 (*GJA3*, *GJA8*) [16,17]. The remainder of underlying mutations occur in a group of functionally diverse genes including those for; heat-shock transcription factor 4 (*HSF*) [18], lens major intrinsic protein (*MIP*) [19], lens intrinsic membrane protein 2 (*LIM2*) [20], transmembrane protein 114 (*TMEM114*) [21], beaded filament structural protein 1 and protein 2 (*BFSP1*, *BFSP2*) [22,23], chromatin modifying protein 4B (*CHMP4B*) [24], Eph-receptor type A2 (*EPHA2*) [25], Tudor domain containing 7 (*TDRD7*) [26], and FYVE and coiled-coil domain containing 1 (*FYCO1*) [27]. Here we have mapped autosomal dominant cataract segregating in a Caucasian American family to chromosome 13q and identified a missense mutation in the gene for gap-junction protein alpha-3 (GJA3), or connexin-46.

## Methods

### Family participants

A 5-generation Caucasian pedigree (family Sh) from the midwestern United States was ascertained through ophthalmic records in the Department of Ophthalmology and Visual Sciences at Washington University School of Medicine, St. Louis MO. Blood samples were obtained from 22 family members including 11 affected individuals. Leukocyte genomic DNA was purified using the Gentra Puregene Blood kit (Qiagen, Valencia, CA), and quantified by absorbance at 260 nm (NanoDrop 2000; Thermo Fisher Scientific, Wilmington, DE). Ethical approval for this study was obtained from the Washington University Human Research Protection Office, and written informed consent was provided by all participants before enrollment in accordance with the tenets of the Declaration of Helsinki, and Health Insurance Portability and Accountability Act (HIPAA) regulations.

### Genotyping and linkage analysis

Microsatellite markers from the National Center for Biotechnology Information (NCBI) combined Généthon, Marshfield, and deCODE genetic linkage maps were genotyped by means of a 4200 DNA analyzer running Gene ImagIR software (Li-Cor, Lincoln, NE) as described [28]. Pedigree and haploptype data were managed using Cyrillic (v.2.1) software (FamilyGenetix Ltd., Reading, UK), and two-point logarithm of the odds (LOD) scores (Z) calculated using the MLINK sub-program from the LINKAGE (5.1) package of programs [29]. Marker allele frequencies were assumed to be equal, and a gene frequency of 0.0001 with a penetrance of 100% were assumed for the disease locus.

### Sequencing analysis

Genomic sequence for *GJA3* was obtained from the Ensemble human genome browser, and gene-specific M13-tailed PCR primers (Table 1) were selected from the NCBI re-sequencing amplicon (RSA) probe database or custom designed (IDT Primer Quest). Genomic DNA (2.5 ng/μl, 20 μl reactions), was amplified (35–40 cycles) in a GeneAmp 9700 thermal cycler using AmpliTaq polymerase (Applied Biosystems, Foster City, CA) and gene-specific primers (10 pmol). Resulting PCR amplicons were either enzyme-purified with ExoSAP-IT (USB Corporation, Cleveland, OH) or gel-purified with the QIAquick gel-extraction kit (Qiagen). Purified amplicons were direct cycle-sequenced in both directions with BigDye Terminator Ready Reaction Mix (version 3.1) containing M13 forward or reverse sequencing primers then ethanol precipitated and detected by capillary electrophoresis on a 3130xl Genetic Analyzer running Sequence Analysis (version 5.2) software (Applied Biosystems), and Chromas (version 2.23) software (Technelysium, Tewantin, Queensland, Australia).

**Table 1:** PCR primers for mutation screening of *GJA3*.

**Primer**	**Location**	**Strand**	**Sequence (5′>3′)**	**Amplicon (bp)**
GJA3-Ex2F1	Codons 128–134	Antisense	CCCGCGACGAGGGATTGT	634
GJA3-Ex2R1	Intron-1	Sense	GACGCTTGCACTTGTGTAGTGCC	** **
GJA3-Ex2F2	Codons 230–235	Antisense	CTGGTCACGCCCTGCTTGAG	512
GJA3-Ex2R2	Codons 77–83	Sense	TTCTGGGCGCTGCAGATCAT	** **
GJA3-Ex2F3	Codons 429–436 (Stop)	Antisense	TAGATGGCCAAGTCCTCCGGTCT	737
GJA3-Ex2R3	Codons 203–210	Sense	TTCATCATCTTCATGCTGGCGGTG	** **
GJA3-Ex2F4	3′-UTR	Antisense	GAGACAGCCCTCAGCGACCA	563
GJA3-Ex2R4	Codons 361–367	Sense	ACTCGCGCACGAGGCTGA	** **

Primer pairs for amplification and sequencing of the coding region (exon-2) of *GJA3* located on 13q.

### Restriction analysis

Allele-specific restriction fragment length analysis was performed on gel-purified PCR amplicons, amplified with primers GJA3-Ex2F1 and GJA3-Ex2R1 (Table 1) using Hsp92 II at 37 °C for 1 h according to the manufacturer’s instructions (Promega, Madison, WI), and digestion products were visualized at 302 nm following electrophoresis in 3% agarose-gels stained with GelRed (Biotium, Hayward, CA). In addition to family Sh, we extended Hsp92 II restriction analysis to include 192 unrelated individuals from the European Collection of Animal Cell Cultures human random control (ECACC-HRC) DNA panel (Sigma, St. Louis, MO) to distinguish the predicted mutation, with 95% confidence, from a polymorphism with 1% frequency as recommended [30].

### Mutation prediction analyses

Missense mutations in GJA3 were evaluated for pathogenicity using three in silico prediction algorithms: Position-Specific Scoring Matrix analysis (PSSM), Sorting Intolerant From Tolerant substitutions (SIFT) [31], and Polymorphism Phenotyping-2 (PolyPhen-2) [32]. GJA3 amino-acid sequences were retrieved from the Entrez protein database, and aligned by means of the ClustalW multiple sequence alignment web server [33]. The hydrophobicity profile of GJA3 was determined by means of the HMMTOP transmembrane topology prediction server [34], and structurally conserved domains located using the Conserved Domain Database (CDD) [35].

## Results

### Linkage analysis

**Figure 1:**
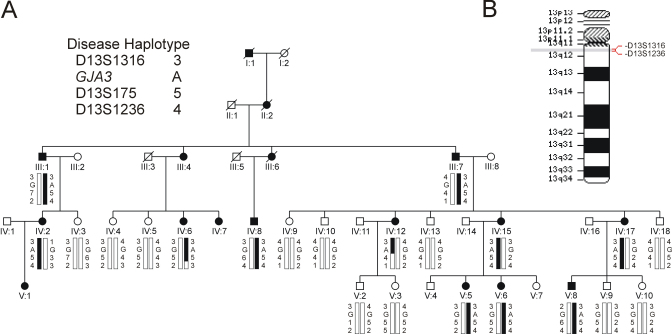
Linkage analysis of autosomal dominant cataract segregating in a 5-generation Caucasian American pedigree (family Sh). **A**: Pedigree and haplotype analysis showing segregation of 3 microsatellite markers on chromosome 13q listed in descending order from the centromere (13p-tel). Squares and circles denote males and females respectively. Filled symbols denote affected status. **B**: Ideogram of chromosome 13 showing the cytogenetic location of the cataract locus.

We studied a 5-generation Caucasian American pedigree (family Sh) segregating autosomal dominant cataract in the absence of other ocular or systemic defects. Autosomal dominant inheritance was supported by the absence of gender bias or skipping of generations. Ophthalmic records described the cataract as congenital in at least four affected individuals (III:I, IV:2, IV:6, and IV:8); however, no slit-lamp images of the lens opacities pre-surgery were available. Twenty-two members of the family (Figure 1), including eleven affected individuals were genotyped with microsatellite markers at 11 candidate loci for autosomal dominant cataract on chromosomes 1q (*GJA8*), 2q (*CRYGC*, *CRYGD*), 3q (*BFSP2*), 11q (*CRYAB*), 12q (*MIP*), 13q (*GJA3*), 16q (*HSF4*), 17q (*CRYBA1*), 19q (*LIM2*), 21q (*CRYAA*), and 22q (*CRYBB1–3*, *CRYBA4*). Following exclusion of 10 of these loci (Z≤-2.0, θ=0.0–0.1), we obtained significant evidence of linkage (Table 2) for marker D13S175 (Z_max_=3.67, θ_max_=0.04) and D13S1316 (Z_max_=2.80, θ_max_=0.0) on 13q11-q12. Haplotyping of the pedigree (Figure 1) detected two affected females, IV:6 and IV:12, who were obligate recombinants at marker D13S1236. Individual IV:12 was also recombinant at D13S175. No other recombinant individuals were detected at the most centromeric marker D13S1316, suggesting that the disease locus lay in the physical interval, D13S1316-(0.17Mb)-D13S175, which contains the strong candidate gene *GJA3*.

**Table 2:** Two-point Lod scores (Z) for linkage between the cataract locus and chromosome 13 markers.

** **	** **	** **	**Z at θ=**						** **	** **
**Marker**	**Mb**	**cM**	**0.00**	**0.05**	**0.10**	**0.20**	**0.30**	**0.40**	**Z_max_**	**θ_max_**
D13S1316	20.68	0.00	2.80	2.50	2.20	1.58	0.95	0.35	2.80	0.00
GJA3 (c.130G>A)	20.71		6.55	6.02	5.46	4.24	2.88	1.35	6.55	0.00
D13S175	20.85	7.40	-∞	3.67	3.46	2.68	1.70	0.66	3.67	0.04
D13S1236	22.70	4.20	-∞	1.48	1.72	1.57	1.11	0.49	1.74	0.12

Z values for markers on 13q listed in physical and genetic distances measured in Mb and cM, respectively, from the short-arm telomere (13p-tel).

### Mutation detection

**Figure 2:**
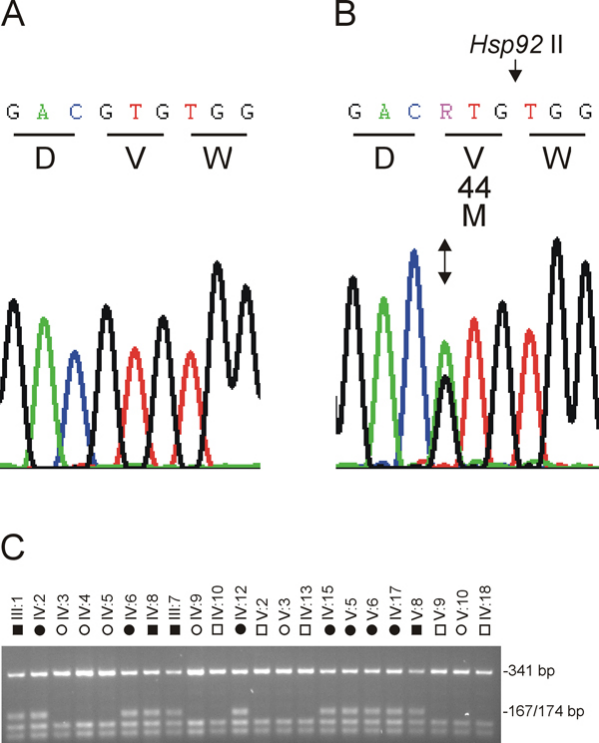
Mutation analysis of *GJA3* in family Sh. **A**: Sequence profile of the wild-type allele showing translation of valine (V) at codon 44 (GTG) in exon 2. **B**: Sequence trace of the mutant allele showing the heterozygous c.130G>A transition (denoted R by the International Union of Pure and Applied Chemistry [IUPAC] code) at the first base of codon 44 (ATG) that is predicted to result in a missense substitution of methionine (M) for valine (p.V44M). **C**: Restriction fragment length analysis on agarose gels showing gain of an Hsp92 II site (5′CATG↓) that co-segregated with affected individuals heterozygous for the mutant A-allele (167/174 bp) but not with unaffected individuals homozygous for the wild-type G-allele (341 bp).

*GJA3* (GeneID: 2700) comprises two exons with exon-2 containing the entire coding region for a 435-amino-acid protein. Sequencing of exon-2 including flanking 5′-intron and 3′-UTR boundaries in two affected relatives detected a heterozygous G-to-A transition (Figure 2) located at position 130 from the first base (A) of the translation start (ATG) codon (c.130G>A). This single nucleotide change was not present in the reference sequence and resulted in the gain of an Hsp92 II restriction site (5′CATG↓). PCR amplification and restriction fragment length analysis confirmed the presence of the heterozygous c.130G>A transition in all affected members of family Sh, and its absence in unaffected relatives (Figure 2). Moreover, when we tested the c.130G>A change as a bi-allelic marker with a notional frequency of 1%, in a two-point LOD score analysis of the cataract locus (Table 2) we obtained further compelling evidence of linkage to *GJA3* (Z_max_=6.55, θ_max_=0). Finally we excluded the c.130G>A transition as a single nucleotide polymorphism (SNP) in a panel of 192 normal unrelated control individuals (384 chromosomes) using allele-specific restriction analysis described in Figure 2 (data not shown). Taken overall our genotype and sequence data strongly suggested that the c.130G>A transition represented a causative mutation rather than a benign SNP in linkage disequilibrium with the cataract phenotype.

### Functional predictions

**Figure 3:**
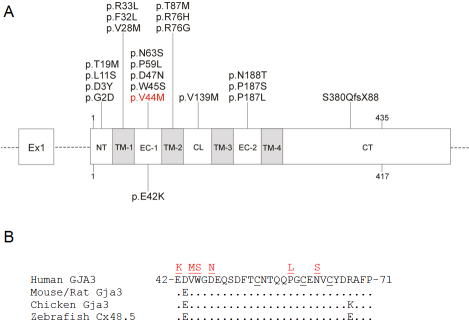
Schematic showing gene structure and protein domains of *GJA3*. **A**: Exon organization and mutation profile of *GJA3*. The entire coding region (435 amino-acids) is located in exon-2. Based on hydrophobicity analysis [34], GJA3 has nine structural domains including: a cytoplasmic N-terminus (NT), 4 transmembrane domains (TM-1 – TM-4); 2 extracellular loops (EC1, EC2), a cytoplasmic loop (CL), and a cytoplasmic C-terminus (CT). The relative locations, with respect to the translation start codon, of the p.V44M mutation and 19 other mutations associated with autosomal dominant cataract in humans are indicated. The rat p.E42K mutation associated with autosomal recessive cataract is also indicated. **B**: Amino-acid sequence alignment of the first extracellular (EC-1) domain (amino-acids 42–71) from human GJA3 and homologs from other species. Dots denote identical amino-acids. Cysteine residues involved in hemi-channel docking are underlined. Missense substitutions are shown in red.

The c.130G>A transition occurred at the first base of codon 44 (GTG>ATG) and was predicted to result in the missense substitution of valine-to-mehionine (p.V44M) at the level of protein translation. The predicted p.V44M substitution represented a relatively conservative amino acid change, with the small non-polar side-group of valine (CH3-CH-CH3) replaced by the larger non-polar side-group of methionine (CH2-CH2-S-CH3). However, cross-species alignment of GJA3 amino-acid sequences revealed that p.V44 is phylogenetically conserved from Zebrafish to man (Figure 3).

Based on the hydrophobicity profile of GJA3, the p.V44M substitution is likely located in the first extracellular (EC-1) loop close to the boundary with the first transmembrane (TM-1) domain (Figure 3). To evaluate the functional consequences of the p.V44M substitution we compared it to all the other missense variations so far identified in *GJA3* using three sequence homology based prediction algorithms (Table 3). PSSM analysis revealed a marked decline in value from +5 to −1 confirming that the predicted p.V44M substitution occurred less frequently than expected in proteins with the conserved connexin superfamily domain (CCD: pfam00029). SIFT analysis gave a score of 0.00 consistent with an “intolerant” amino-acid change, and PolyPhen-2 analysis gave a score of 1.00 consistent with a “probably damaging” change, further raising the likelihood of GJA3 dysfunction.

**Table 3:** Summary of mutations/variations found in *GJA3* (exon 2) associated with autosomal dominant and age-related forms of cataract.

**DNA change**	**Coding change**	**PSSM wt/mut**	**SIFT**	**PolyPhen2**	**Protein domain**	**Cataract phenotype**	**Origin**	**References**
c.-39C>G	-	-	-	-	-	Age-related nuclear	China	[41]
c.5G>A	p.G2D	-	0.00	1.000	NH_2_-Term	Nuclear pulverulent and posterior polar	China	[48]
c.7G>T	p.D3Y	+6/-4	0.00	1.000	NH_2_-Term	Zonular pulverulent	Honduras	[49]
c.32T>C	p.L11S	+6/-4	0.00	1.000	NH_2_-Term	“Ant egg”	Denmark	[50]
c.56C>T	p.T19M	+8/-3	0.00	1.000	NH_2_-Term	Posterior polar	India	[51]
c.82G>A	p.V28M	+6/-1	0.00	0.970	TM-1	“Total, anterior capsular, cortical”	India	[40]
c.96C>A	p.F32L	+9/-1	0.00	0.999	TM-1	Nuclear pulverulent	China	[52]
c.98G>T	p.R33L	+8/-4	0.00	1.000	TM-1	Granular embryonal	India	[53]
c.130G>A	p.V44M	+5/-1	0.00	1.000	EC-1	Nuclear	China	[36]
c.130G>A	p.V44M	+5/-1	0.00	1.000	EC-1	?	USA	This study
c.134G>C	p.W45S	+12/-4	0.00	1.000	EC-1	Nuclear	China	[54]
c.139G>A	p.D47N	+8/-1	0.01	1.000	EC-1	Nuclear	China	[55]
c.176C>T	p.P59L	+9/-5	0.00	1.000	EC-1	Nuclear punctate	USA	[37]
c.176C>T	p.P59L	+9/-5	0.00	1.000	EC-1	?	Denmark	[38]
c.188A>G	p.N63S	+8/+1	0.00	0.833	EC-1	Variable pulverulent	UK	[17]
c.226C>G	p.R76G	+8/-4	0.00	0.961	TM-2	Total	India	[40]
c.227G>A	p.R76H	+8/-2	0.00	1.000	TM-2	Nuclear lamellar pulverulent	Australia	[39]
c.227G>A	p.R76H	+8/-2	0.00	1.000	TM-2	?	Denmark	[38]
c.260C>T	p.T87M	+6/-2	0.00	1.000	TM-2	“Pearl-box”	India	[56]
c.415G>A	p.V139M	-	0.07	0.818	CL	Age-related cortical	China	[41]
c.560C>T	p.P187L	+8/-2	0.00	0.999	EC-2	Zonular pulverulent	UK	[57]
c.559C>T	p.P187S	+8/-2	0.00	0.961	EC-2	Nuclear pulverulent	China	[58]
c.563A>C	p.N188T	+6/-2	0.02	0.931	EC-2	Nuclear pulverulent	China	[59]
c.1137insC	p.S380QfsX88	-	-	-	COOH-Term	Punctate	UK	[17]

SIFT scores <0.05 are intolerant and scores ≥0.05 are tolerant. PolyPhen-2 scores >0.85 are probably damaging and scores 0.15–0.85 are possibly damaging.

## Discussion

Here we have identified a heterozygous transition (c.130G>A) in exon-2 of *GJA3* co-segregating with autosomal dominant cataract linked to chromosome 13q in a Caucasian American family. This missense mutation was predicted to result in a conservative p.V44M substitution in the first extracellular domain of GJA3 with damaging effects on protein function. Recently, the same *GJA3* mutation was detected by candidate-gene sequencing in a Han Chinese family segregating autosomal dominant cataract described as central nuclear with punctate cortical opacities [36]. However, no supporting linkage analysis or functional studies were performed. Our data confirm recurrent association of the p.V44M substitution in GJA3 with autosomal dominant cataract linked to 13q.

Currently, at least 19 different heterozygous coding mutations in *GJA3* (Table 3) have been detected in 22 families worldwide making it one of the most frequently mutated genes associated with autosomal dominant cataract. The resulting opacities are usually described as nuclear or zonular/lamellar often with a pulverulent (dustlike) or punctate appearance. All but one of the known coding mutations in *GJA3* are missense substitutions (Table 3) that are located toward the NH_2_-terminal end of the protein containing the conserved connexin domain (CCD: pfam00029) and the gap-junction channel protein cysteine-rich or conexin_CCC domain (CCD: pfam10582). Five of these missense substitutions, including p.V44M identified here, are believed to be located in the first extracellular (EC-1) domain of GJA3 (Figure 3). In addition to p.V44M, two other missense mutations in GJA3 are recurrent with autosomal dominant cataract. A p.P59L substitution in the first extracellular domain has been reported in American and Danish families [37,38], whereas, a p.R76H substitution in the second transmembrane domain has been detected in Australian and Danish families [38,39]. Furthermore, two other valine-to-methionine substitutions have been reported in GJA3. A p.V28M change in the first transmembrane domain has been associated with autosomal dominant cataract in an Indian family [40], and a p.V139M change in the cytoplasmic loop has been associated with age-related cortical cataract in a Chinese population [41]. Interestingly, both p.V28M and p.V44M were predicted to be probably damaging to GJA3 function, whereas, p.V139M was predicted to be a benign or possibly damaging variant (Table 3).

So far no mutations in the mouse *Gja3* gene have been associated with spontaneous or chemically/radiologically induced forms of cataract. By contrast a homozygous missense substitution (p.E42K) in rat Gja3 underlies a spontaneous form of autosomal recessive nuclear cataract in the SHRSPwch1.9^Cat^ strain [42]. Knockout mice lacking Gja3 as a result of gene disruption also develop nuclear cataract with severity of lens opacification influenced by genetic background [43,44]. However, hemizygous loss of *Gja3* does not elicit cataract in mice.

Mouse Gja3 has been proposed to function in gap-junction coupling of lens fiber cells [45]; the primary target cells for cataract. In addition, Gja3 has been shown to form active hemi-channels in dissociated mouse lens fiber cells [46]. Structure-function prediction algorithms show that 18 of 19 reported missense substitutions in GJA3 are likely to be damaging to protein function (Table 3). Functional expression studies of one GJA3 missense mutant, p.N63S, in *Xenopus* oocytes revealed that it exhibited impaired hemi-channel activity in single oocytes, and failed to elicit gap-junction coupling in paired oocytes [47]. While p.N63S is located in the conserved tri-cysteine motif within the first extracellular domain of GJA3, p.V44M identified here and p.E42K identified in the rat are located near the boundary between the first extracellular domain and the first transmembrane domain (Figure 3). Both p.V44M and p.N63S are associated with autosomal dominant cataract, whereas, p.E42K is associated with autosomal recessive cataract. In general mutations underlying autosomal dominant phenotypes result in deleterious gain-of-function mechanisms, whereas, those underlying autosomal recessive phenotypes elicit loss-of-function mechanisms. Further detailed functional expression studies will be required to elucidate the precise pathogenic mechanisms that link *GJA3* mutations with cataract.
